# Efficient learning-based blur removal method based on sparse optimization for image restoration

**DOI:** 10.1371/journal.pone.0230619

**Published:** 2020-03-27

**Authors:** Haoyuan Yang, Xiuqin Su, Songmao Chen, Wenhua Zhu, Chunwu Ju

**Affiliations:** 1 Xi’an Institute of Optics and Precision Mechanics, Chinese Academy of Sciences, Xi’an, Shaanxi, China; 2 University of Chinese Academy of Sciences, Beijing, China; Chongqing University, CHINA

## Abstract

In imaging systems, image blurs are a major source of degradation. This paper proposes a parameter estimation technique for linear motion blur, defocus blur, and atmospheric turbulence blur, and a nonlinear deconvolution algorithm based on sparse representation. Most blur removal techniques use image priors to estimate the point spread function (PSF); however, many common forms of image priors are unable to exploit local image information fully. In this paper, the proposed method does not require models of image priors. Further, it is capable of estimating the PSF accurately from a single input image. First, a blur feature in the image gradient domain is introduced, which has a positive correlation with the degree of blur. Next, the parameters for each blur type are estimated by a learning-based method using a general regression neural network. Finally, image restoration is performed using a half-quadratic optimization algorithm. Evaluation tests confirmed that the proposed method outperforms other similar methods and is suitable for dealing with motion blur in real-life applications.

## Introduction

Imaging systems suffer from several types of image degradations. Motion blur is a common phenomenon, as are defocus blur and atmospheric turbulence blur. All of them reduce the image quality significantly. Therefore, it is essential to develop methods for recovering approximated latent images from blurry ones in order to increase the performance of imaging systems. Such methods will find wide applicability in various fields. However, the issue of blur removal is a notoriously ill-defined inverse problem that has perplexed scholars for decades [1]. A point spread function (PSF) can be used to describe image blur. The PSF models how the imaging system captures a point source or object and describes how the point spreads across an image. Generally, the PSF can be transformed into a parametric model [2], with the parameters being the motion length, defocus radius, and turbulence degree [3].

For estimating the PSF, Fergus et al. [4] proposed a method that combines the gradient domain of natural images with the maximum a posteriori (MAP) method. Xu et al. [5] introduced a sparsity function with an $l_{0}$-norm constraint, while Pan et al. [6] estimated the blur kernel from the dark channel of the blurry images. These estimation techniques perform well when dealing with hand-drawn PSFs. However, in real-life situations, the blur models are often known. For example, when monitoring targets on a conveyor belt with a fixed camera, the PSF can be modeled based on the motion length during the exposure time. Similarly, with respect to space exploration, the atmospheric turbulence can be modeled by a Gaussian function, whose variance indicates the blur degree. Defocus blur can be modeled based on the defocus radius. It is more convenient and practical to solve a parameter identification problem than to estimate the PSF. Given this fact, Jalobeanu et al. [7] used the maximum likelihood estimator (MLE) on the entire dataset available to estimate the parameters for a Gaussian model. Yin and Hussain [8] combined the non-Gaussianity measures for independent component analysis to estimate the parameters for blur models. Dash and Majhi [9] suggested a radial basis function neural network with image features based on the magnitude of Fourier coefficients to estimate the motion lengths. Yan and Shao [10] proposed a supervised deep neural network to classify the blur type and adopted the expected patch log likelihood method [11] to restore the latent image. Further, Kumar et al. [12] used the Tchebycheff moment to estimate the Gaussian variance for turbulence blurs.

With a known PSF, the latent image can be restored using inverse filters or some other nonblind deconvolution method. Levin et al. [13] proposed a hyper-Laplacian prior and adopted the iterative reweighted least squares (IRLS) algorithm to solve the optimization problem. Joshi et al. [14] adopted the IRLS algorithm for local color statistics and hyper-Laplacian priors in order to perform deblurring and denoising. Wang et al. [15] introduced a deconvolution method based on the total variation and employed the half-quadratic minimization (HQM) algorithm, which was originally proposed by Geman and Yang [16], to solve the nonconvex problem.

Simultaneous estimation of both the PSF and the latent image is a nonconvex issue that always results in a nonblur solution [17]. A feasible blind image deblurring framework is to estimate the PSF and the latent image alternately [4, 6, 18]. However, this framework requires prior knowledge of both the image and the PSF. In addition, because this procedure would be sensitive to noise, the image edges must be reconstructed during each iteration [5] before the next step. In addition, in order to avoid local minima, a coarse-to-fine technique must also be used during the alternating optimization process. All the disadvantages mentioned above make the deblurring procedure time-consuming. Furthermore, most existing approaches are designed for random hand-drawn blurs. However, in real-world situations, the blur model would always be known. In the case of target detection on a conveyor belt, the PSF only depends on the motion length. From this viewpoint, the PSF estimation procedure in the existing methods can be simplified, and the speed of the deblurring algorithm can be increased.

In this paper, a new deblurring framework consisting of two stages is proposed. First, a blur feature is used to estimate the model parameters via a general regression neural network (GRNN) in order to determine the PSF. Next, a deconvolution algorithm based on sparse representation is proposed for latent image restoration. The main contributions of this paper can be summarized as follows:

- Three common types of blur models are discussed and a blur feature based on autocorrelation of the image gradient domain is proposed. Simulations show that its amplitude rises with an increase in the blur degree. Then, more than ten thousand natural image samples were artificially blurred for feature extraction to generate the training dataset for the GRNNs.
- A learning-based parameter estimation scheme is proposed. With the help of the GRNNs, the blur model parameters for input blurry images can be estimated based on their blur features. Then, their PSFs can be constructed.
- A nonblind deblurring algorithm based on sparse representation is presented. Its target image is constrained by an $l_{p}$ quasi-norm in the cost function.
- The solutions for different *p* values are discussed and their performances on some typical *p* values are analyzed. Further, the proposed deblurring method is compared with several related blur removal approaches.

The remainder of this paper is organized as follows: Section 2 introduces the imaging system and the parametric model for the three most common blurs. Next, the proposed blur feature is described. Section 3 describes the proposed deblurring framework, including the properties of the GRNN and the restoration algorithm. The results of the simulations and comparative analysis performed are described in Section 4. Section 5 summarizes the study and presents concluding remarks.

### Blur models and features

#### Imaging system

**Fig 1:**
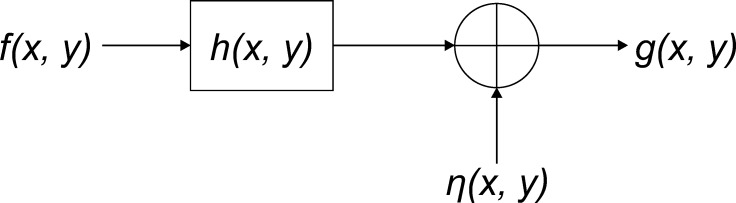
Image blurring in imaging system.

An imaging system can generally be regarded as a linear-translation-invariant system [19], and the image blurring procedure, shown in Fig 1, can be described as follows:

g(x,y)=h(x,y)*f(x,y)+η(x,y) (1)

where *f*(*x*,*y*) and *g*(*x*,*y*) represent the original image and the observed blurry image, respectively; *h*(*x*,*y*) is the PSF; *η*(*x*,*y*) is the additive noise generated during image acquisition or transmission; *x* and *y* represent the coordinates of a digital image; and the symbol “*” represents the convolution operator. For simplicity, we can also use bold notation (such as **f** and **h**) to represent the image and PSF, respectively.

### Parametric model of blurs

**Fig 2:**
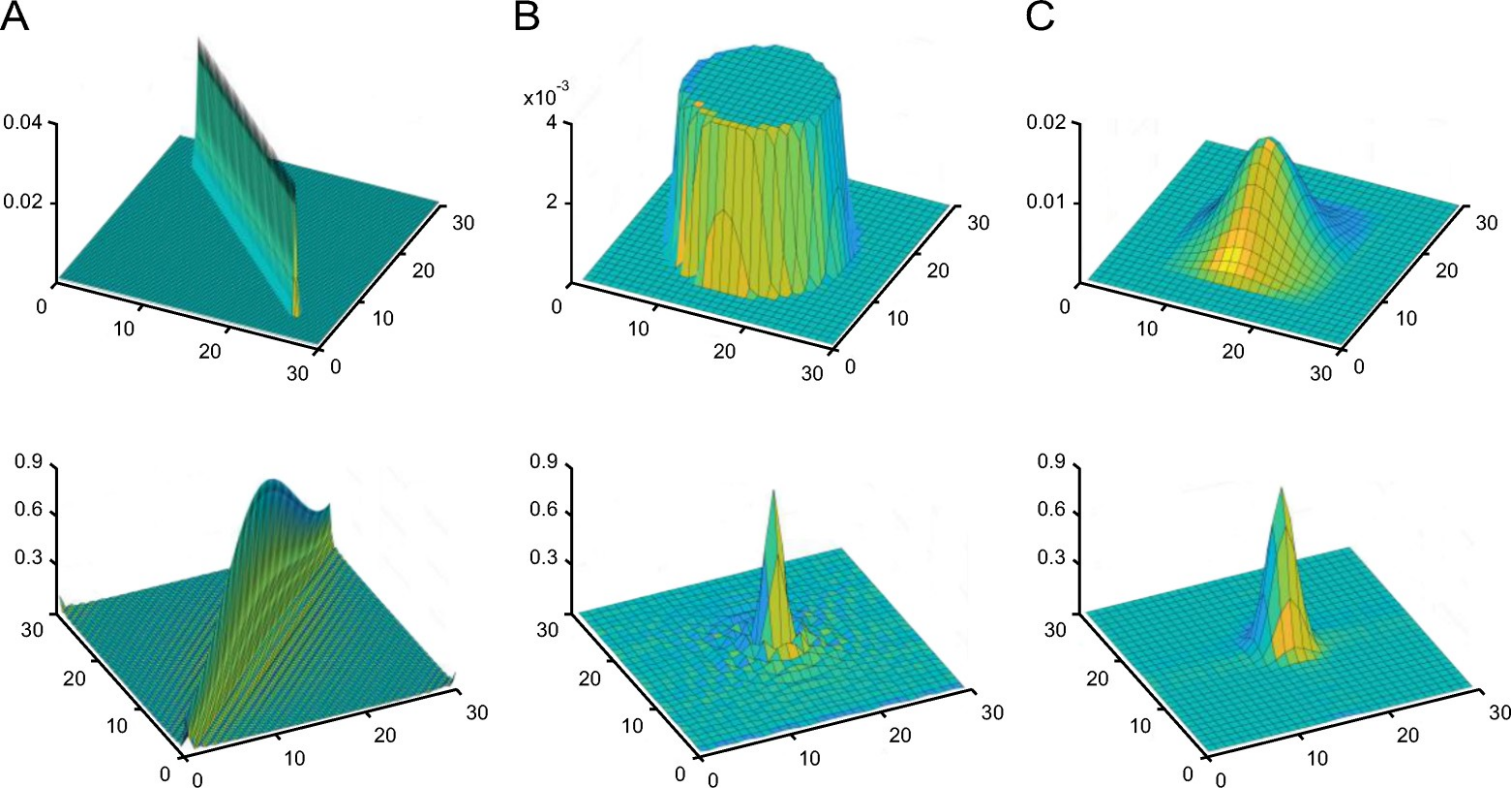
Examples of the PSF (first row) and its frequency domains (second row). Left: Motion blur with (*L*,*Φ*) = (30,45°) in Eq (2). Middle: Defocus blur with *r* = 9 in Eq (3). Right: Gaussian blur with *σ* = 3.5 in Eq (4).

*a) Linear motion blur* [20]. Relative movement between the camera and the target when the exposure time is insufficiently small results in linear motion blur. Its PSF can be modeled as follows:

$$h\left(x,y;L,\Phi \right)=\{\begin{array}{cc} L^{-1}, & \sqrt{x^{2}+y^{2}}\leq \frac{L}{2},\Phi =-\frac{x}{y}\\ 0 & otherwise \end{array}$$ (2)

where *L* represents the motion length and *Φ* is its orientation. An example of linear motion blur is shown in Fig 2A.

*b) Defocus Blur* [21]. In the case of defocusing, blurring arises because of the optical system of the lens. The path followed by light through the lens depends on its wavelength. However, natural light consists of components with several different wavelengths. This results in physical limitations with respect to the construction of lenses that prevent the camera from producing perfectly sharp images, leading to defocus blur. The extent of blur that is visible in an image is a function of the lens aperture:

$$h\left(x,y;r\right)=\{\begin{array}{cc} \frac{1}{\pi r^{2}}, & {\left(x-x_{0}\right)}^{2}+{\left(y-y_{0}\right)}^{2}\leq r^{2}\\ 0, & otherwise \end{array}$$ (3)

where the radius, *r*, determines the blur extent. Further, (*x*_0_,*y*_0_) is the center of the PSF. This is depicted in Fig 2B.

*c) Atmospheric turbulence blur* [7]. The variations in the heat, pressure, and wind velocity in the atmosphere result in small-scale, irregular air motions characterized by winds, which vary in speed and direction. These have a determining effect on light propagation and cause atmospheric turbulence blur, which is also known as Gaussian blur. This type of blur usually occurs during remote sensing. Generally, a low-pass Gaussian filter [3, 22, 23] can be used to model it:

$$h\left(x,y;\sigma \right)=\frac{1}{2\pi \sigma }\exp\left\{-\frac{{\left(x-x_{0}\right)}^{2}+{\left(y-y_{0}\right)}^{2}}{2\sigma^{2}}\right\}$$ (4)

where *σ* indicates the blur degree and (*x*_0_,*y*_0_) is the center of the PSF. Since Eq (4) is an infinite impulse response filter, proper truncation and normalization are necessary in practice. Normally, the support domain is set as [*x*_0_−3*σ*,*x*_0_+3*σ*]×[*y*_0_−3*σ*,*y*_0_+3*σ*], since 99.7% of the energy is contained in this region; here, × is the Cartesian product. Fig 2C show an example of the Gaussian blur model.

### Blur features

Natural images are diverse, and their statistical characteristics vary significantly. Nevertheless, in recent years, an increasing number of studies have indicated that the gradient domains of natural images share common features and that their histograms tend to have sharp tops and long tails [24]. Generally, a digital image can be treated as a discrete binary function mathematically, and its gradient domain can be represented based on the pixel difference. In practice, image edge detection operators [3, 25] such as the Roberts and Prewitt operators are used commonly for detecting the first-order derivatives while the Sobel and Laplacian operators are used for the second-order derivatives.

In this study, inspired by the properties of image edges, a blur feature based on the autocorrelation of blurry images in the gradient domain is proposed. By definition, autocorrelation is the correlation of a signal with a delayed copy of itself as a function of the delay [26]. It is a useful tool for measuring the similarity between a signal and its shifted versions. A digital image can be treated as a two-dimensional (2D) real signal and its gradient domain *e*(*x*,*y*) as a binary function. Thus, its autocorrelation *R*(*x*,*y*) can be described as

$$R\left(x,y\right)=\underset{m\in Z_{x}}{\sum }\underset{n\in Z_{y}}{\sum }e\left(m,n\right)e\left(m-x,n-y\right)$$ (5)

where *Z*_*x*_ and *Z*_*y*_ are the support domains of *e*(*x*,*y*).

In addition, based on the conjugate symmetry of real signals [27, 28], the computation of Eq (5) can be accelerated with the help of the fast Fourier transform (FFT) and inverse fast Fourier transform (IFFT). Therefore, the autocorrelation of the image edge, *e*(*x*,*y*), can be simplified to

$$R=F^{-1}\left(|F\left(e\right){|}^{2}\right)$$ (6)

where R and **e** represent the matrix form of *R*(*x*,*y*) and *e*(*x*,*y*), respectively, for brevity, and F(⋅) and $F^{-1}(\cdot )$ are the FFT and IFFT operators. Further, |⋅|^2^ represents the element-wise square of the modulus of the former.

Since the value range of image edges will vary widely, their blur features should be adjusted on a notionally common scale. Feature scaling [29], also known as unity-based normalization, is a technique that is used to bring all values within the range [0,1]. Hence, the normalized blur feature, $R_{norm}$, can be described as follows:

$$R_{norm}=\frac{R-R_{min}}{R_{max}-R_{min}}$$ (7)

where $R_{max}$ and $R_{min}$ are the maximum and minimum values of R.

**Fig 3:**
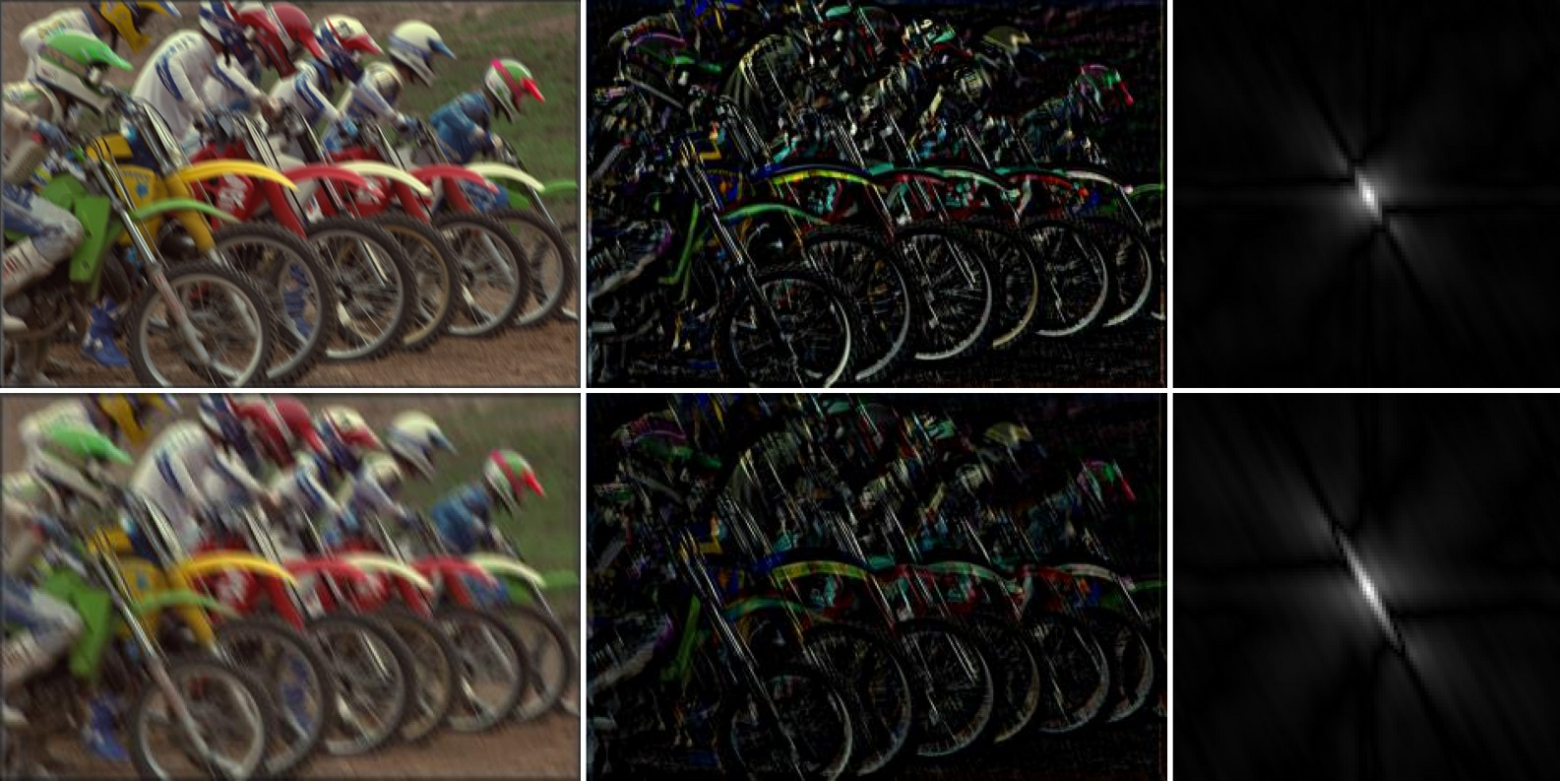
Examples of the blur feature in motion blur. Left: Motion blur of the “bikes.bmp” image with *L* =10 (first row) and *L* = 20 (second row) and orientation *Φ* = 120°. Middle: Gradient domain. Right: Amplitudes of the blur features.

**Fig 4:**
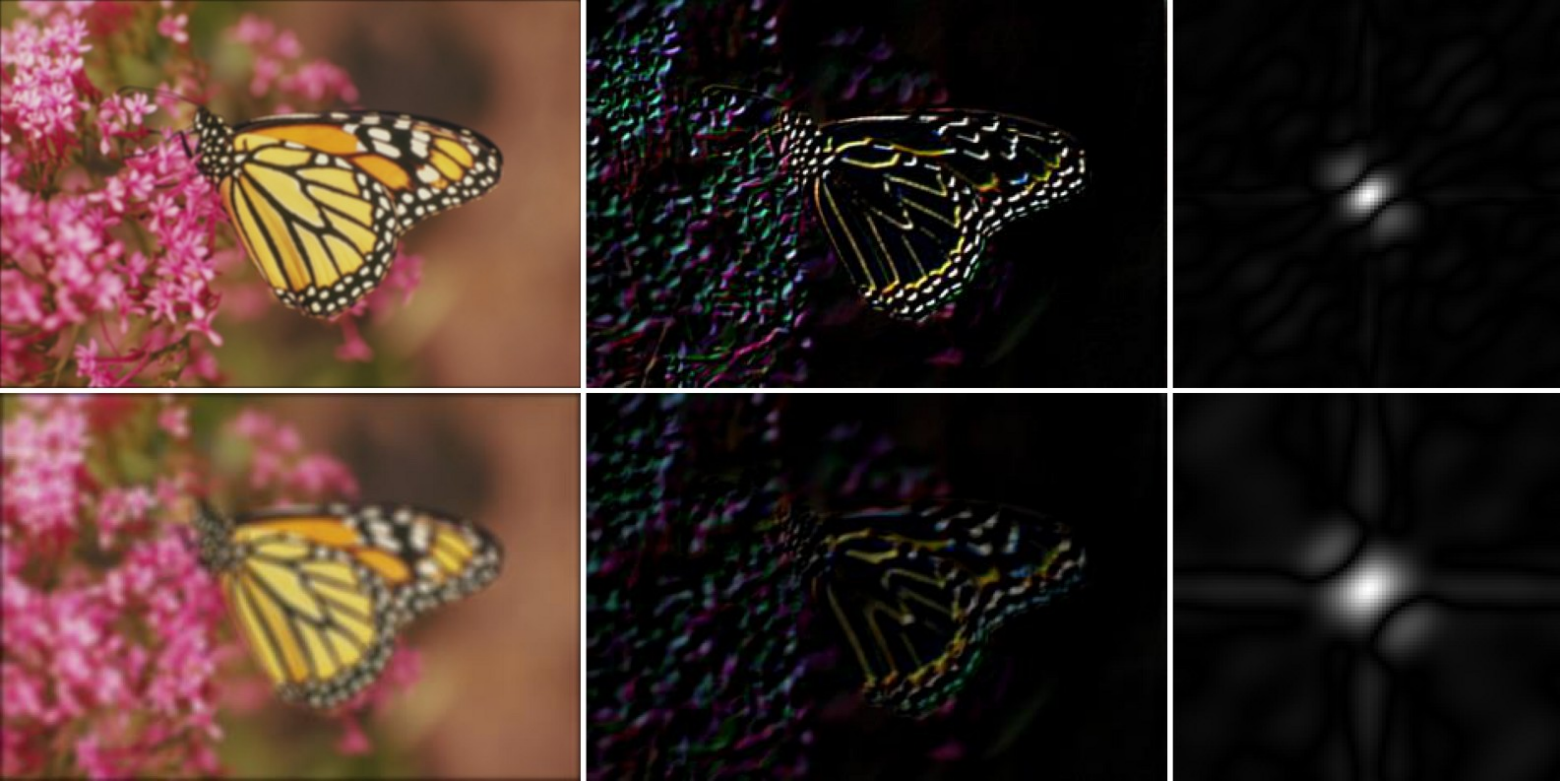
Examples of the blur feature in defocus blur. Left: Defocus blur of the “monarch.bmp” image with *r* = 3 (first row) and *r* = 3 (second row). Middle: Gradient domain. Right: Amplitudes of the blur features.

**Fig 5:**
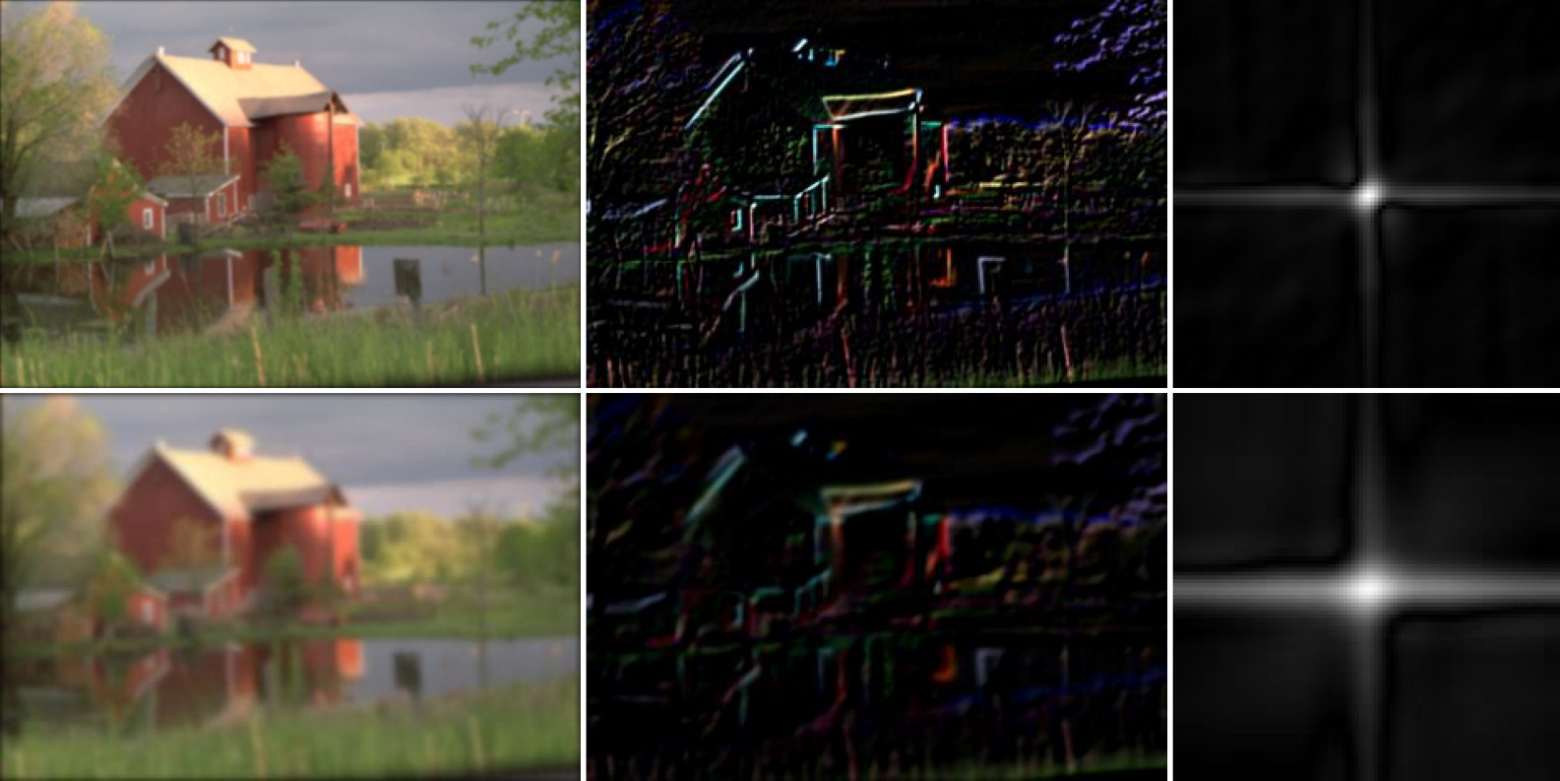
Examples of the blur feature in atmospheric turbulence blur. Left: Atmospheric turbulence blur of the “house.bmp” image with *σ* = 2.0 (first row) and *σ* = 4.8 (second row). Middle: Gradient domain. Right: Amplitudes of the blur features.

In Figs 3–5, the left column shows examples of natural images that were blurred artificially by motion blur, defocus blur, and Gaussian blur using different parameters. Their gradient domain versions were extracted by using the Sobel operator both in the horizontal and the vertical directions. The image edges, which were obtained by adding them, are shown in the middle column. As can be seen, with an increase in the image blurriness, the information contained in the edges decreases. The right column shows the amplitude of the blur feature described in Eqs (5)–(7). It can be seen from Fig 3 that the features of the motion-blurred images retain the orientation of the PSF, but the length of the shape of the blur features increases with the motion length. In the case of the defocus blur, the amplitude range of the feature increases with the defocus radius, *r*, as shown in Fig 4. Finally, the feature of the Gaussian blur in Fig 5 expands with an increase in parameter *σ*.

Note that, in Figs 3–5, because the size of the blur features depends on the size of the images, according to Eqs (5)–(7), and most of the amplitude energy is located near the center of the blur feature, their boundaries have been truncated to ensure that the blur features are all the same size.

### The proposed learning-based blur removal method

#### General regression neural network

A GRNN is a probabilistic neural network (PNN), and was first proposed by Specht [30]. It can converge to the underlying function of the data with only a few training samples available, and its spreading rate is the only additional parameter that requires adjustment by the user. This makes GRNNs a very useful tool in areas such as regression, prediction, and classification.

**Fig 6:**
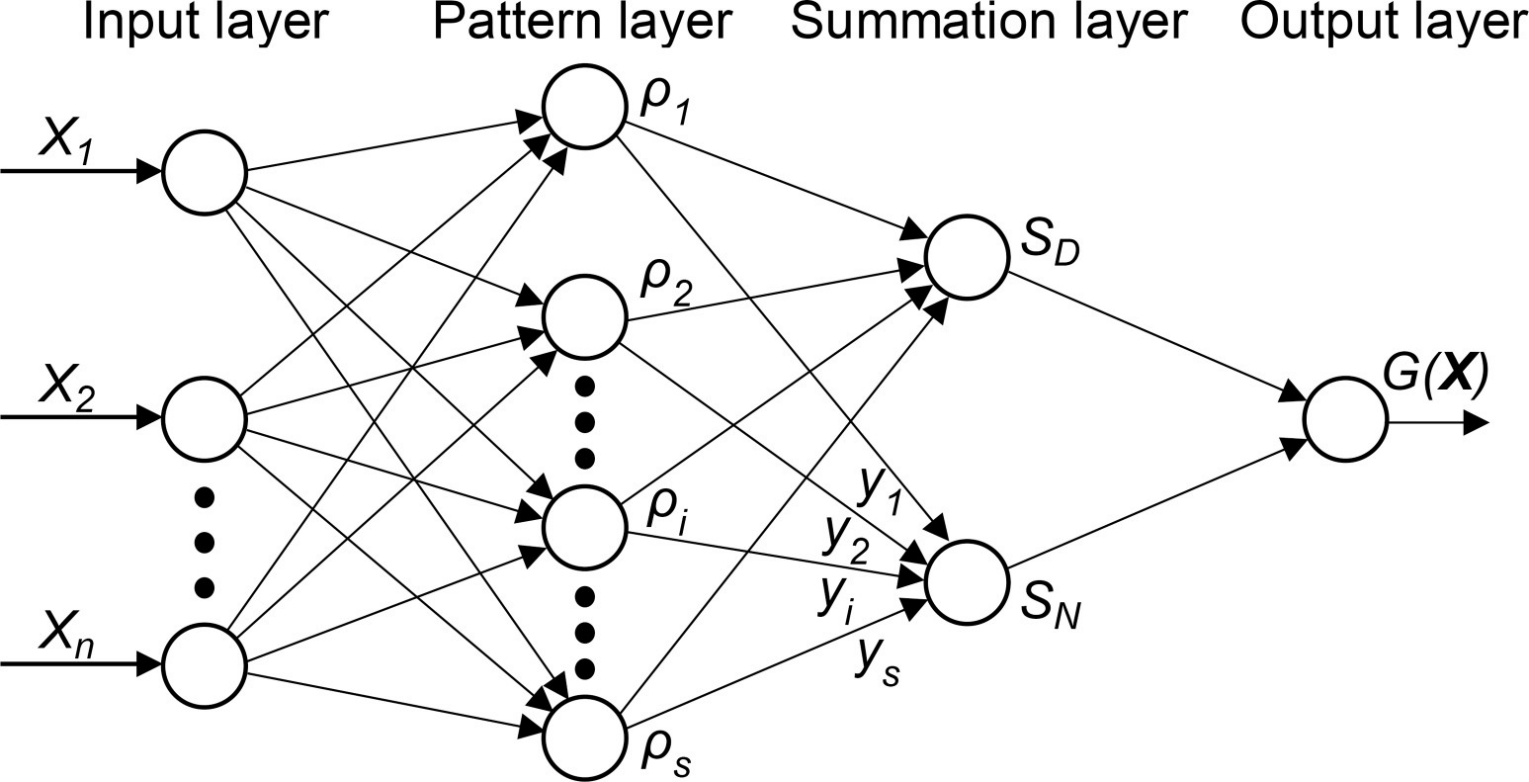
Basic structure of a GRNN.

A GRNN is composed of an input layer, a pattern layer, a summation layer, and an output layer [31]. Its overall block diagram is shown in Fig 6. In the input layer, ***X*** = (*X*_1_,*X*_2_,⋯*X*_*n*_) represents the input data of the GRNN *G*(***X***).

In the pattern layer, the number of units, *s*, is equal to the length of training data (*x*_1_,*x*_2_,⋯*x*_*s*_) and (*y*_1_,*y*_2_,⋯*y*_*s*_). The activation function of each unit, *i*, is given by

$$\rho_{i}=\exp\left\{-\frac{{\left(X-x_{i}\right)}^{T}\left(X-x_{i}\right)}{2\delta^{2}}\right\},\;i=1,2,\cdots s$$ (8)

where *δ* is the spreading rate, and **T** represents the transpose.

In the summation layer, $S_{D}=\sum_{i=1}^{s}\rho_{i}$ and $S_{N}=\sum_{i=1}^{s}\rho_{i}y_{i}$ need to be determined. Their ratio yields the final output, *G*(***X***) = *S*_*N*_/*S*_*D*_.

Taking another perspective, a GRNN is a generalization of a radial basis function network (RBFN) and a PNN. In the pattern layer, the activation function in Eq (8) is similar to the radial basis function kernel (Gaussian kernel), and it uses the training sample *x*_*i*_ as the mean value of each unit. In the output layer, *S*_*N*_/*S*_*D*_ indicates the probability of how well the training sample can represent the prediction position. In practice, the GRNN outperforms the RBFN as well as traditional back propagation neural networks in predictions, as the former only involves a one-pass learning scheme instead of repetitive iterations during the training process [30]. Therefore, the advantages of the GRNN are fast learning and convergence, even though the number of inputs is very high.

#### Framework of the deblurring method

**Fig 7:**
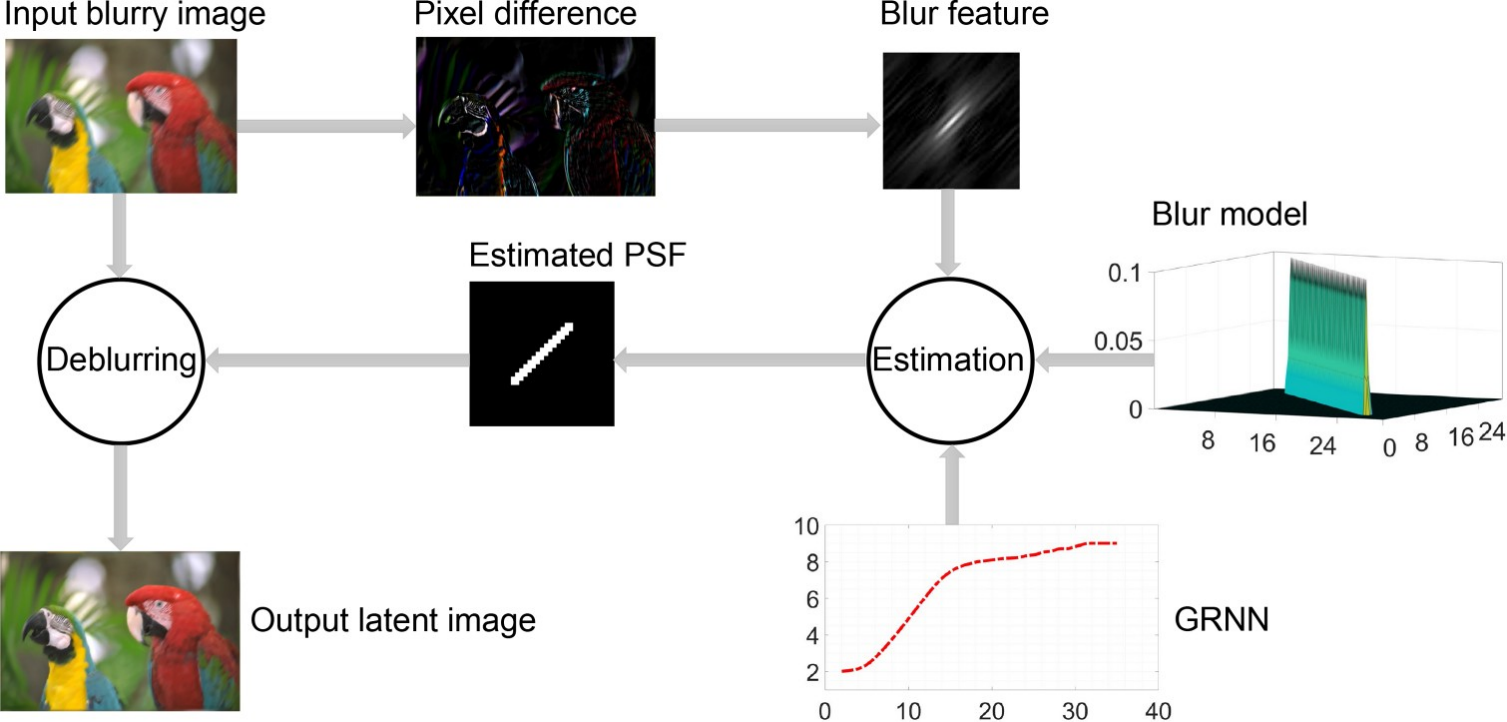
Framework of the proposed method. The “Estimation” and “Deblurring” processes are described in Algorithms 1 and 2, respectively.

The principal framework of the proposed deblurring method is shown in Fig 7. It consists of two primary steps. The first is a learning-based parameter estimation procedure while the second involves the use of a nonlinear deblurring algorithm.

In order to estimate the blur parameters efficiently, more than ten thousand natural images were artificially blurred using the three above-mentioned blur models and different parameters, and their blur features were extracted using Eq (7). The Frobenius norms [32] of these features, $∥R_{norm}{∥}_{F}$, were taken as the inputs to train a GRNN for each blur model. In this manner, for a given blurred image, the PSF could be estimated from its blur feature while using the GRNN. Next, the latent image was restored via a nonblind blur removal algorithm, which is discussed in the following subsection. The main steps for parameter estimation are shown in Algorithm 1.

Algorithm 1: Parameter Estimation

Input: (1) Observed blurry image: **g**;

        (2) GRNN: *G*(***X***);

Output: Deblurring result: **f**;

1: Extract the edges, **e**, from the input blurry image, **g**;

2: Calculate the blur feature, $R_{norm}$, using Eq (7);

**3:** Estimate the blur model parameter, *Y*, using the network, $Y=G(∥R_{norm}{∥}_{F})$;

**4:** Generate the PSF, **h**, from the estimated *Y*;

**5:** Restore the latent image, **f**, using Algorithm 2;

6: return **f**;

#### Latent image restoration

Once the PSF has been acquired, a variety of nonblind deconvolution methods can be used to recover the latent image. Wiener filtering and the Lucy-Richardson method are frequently used. These deblurring techniques were proposed decades ago based on the MLE. However, they both have the disadvantage of being sensitive to any incorrect PSF estimate, and the ringing effect [33, 34] is unavoidable. In recent years, the theory of sparse representation and machine learning have been introduced in the field of image restoration [35–37]. Based on a statistical analysis of natural images, several studies have shown that the image gradients tend to have a heavy-tailed distribution [38, 39]. The most commonly used form of this distribution is the hyper-Laplacian model [13, 38]. For each element of the image gradient *∇***f**_*i*_, the hyper Laplacian model can be expressed by the joint distribution of **f** as follows:

$$P\left(f\right)=\underset{i}{\prod }\exp\left\{-\alpha {\left|\nabla_{*}f_{i}\right|}^{p}\right\},\;p\in \left[0.5,0.8\right]$$ (9)

where *∈{*x*,*y*} represents the gradient in two orientations and *α* signifies the slope of the exponential function. From the perspective of MAP estimation, the image prior is turned into a regularization term in the logarithm cost function [17]. Let ‖⋅‖_*p*_ represent a quasi-norm, which is defined as

$$∥f{∥}_{p}=\underset{i}{\sum }{\left|f_{i}\right|}^{p}.$$ (10)

In this study, the cost function was defined as follows:

$$\hat{f}=\arg\;\underset{f}{\min}∥f*h-g{∥}_{2}^{2}+\alpha ∥\nabla f{∥}_{p}$$ (11)

where *∇***f** is short for (*∇*_*x*_**f**, *∇*_y_**f**). The first term on the right-hand side of the equation is a data fidelity term to ensure the best approximation of the original image while the second term is a constraint on the image gradient.

Because the optimization problem described in Eq (11) is nonlinear, traditional descent methods are ineffective because of their slow convergence. Fortunately, inspired by the half-quadratic penalty method [15, 16, 40], which can alternately optimize the $l_{p}$-based expression in Eq (11) when 0<*p*≤1, the problem can be simplified to an alternating optimization problem:

$$\underset{f,u}{\min}∥f*h-g{∥}_{2}^{2}+\beta ∥\nabla f-u{∥}_{2}^{2}+\alpha ∥u{∥}_{p}$$ (12)

where *β* is an intermediate coefficient that is varied during the alternating scheme. The solution of Eq (12) converges to that of Eq (11) as *β*→∞, and the optimization procedure can be described by the two subproblems given below:

(1) Update **u**. For a given **f**, Eq (12) becomes an $l_{p}$-based constrained optimization problem,

$$\hat{u}=\arg\;\underset{u}{\min}∥u{∥}_{p}+\frac{\beta }{\alpha }∥\nabla f-u{∥}_{2}^{2}.$$ (13)

(2) Update **f**. For a given **u**, the minimization problem is reduced to an $l_{2}$-norm constrained optimization problem; this is also known as least-squares minimization,

$$\hat{f}=\arg\;\underset{f}{\min}∥f*h-g{∥}_{2}^{2}+\beta ∥\nabla f-u{∥}_{2}^{2}.$$ (14)

For the **u** subproblem, the solution of Eq (13) varies for different *p* values. In particular, when *p* = 1, it becomes a total variation problem, whose analytical solution can be obtained by the soft thresholding algorithm [15],

$$\hat{u}=\mathrm{sgn}\left(u\right)\max\left\{0,\left|u\right|-T\right\}$$ (15)

where sgn(⋅) is the signum function and threshold *T* = *α*/(2*β*).

In general, for a given *p*, the minimization problem can be solved by setting the derivative of Eq (13) to zero. Thus, for the *i*th element of **u** and *∇***f**, we get

$$p{\left|u_{i}\right|}^{p-1}\mathrm{sgn}\left(u_{i}\right)+\frac{2\beta }{\alpha }\left(u_{i}-f_{i}\right)=0.$$ (16)

In particular, when *p* = 0.5, Eq (16) can be simplified to a cubic function,

$$u_{i}^{3}-2f_{i}u_{i}^{2}+f_{i}^{2}u_{i}-{\left(\frac{\alpha }{4\beta }\right)}^{2}\mathrm{sgn}\left(u_{i}\right)=0$$ (17)

which can be solved using Cardano’s formula [41].

When *p* = 2/3, Eq (16) can be expanded as a quartic function,

$$u_{i}^{4}-3f_{i}u_{i}^{3}+3f_{i}^{2}u_{i}^{2}-f_{i}^{3}u_{i}-{\left(\frac{\alpha }{3\beta }\right)}^{3}=0$$ (18)

which can be solved using Ferrari’s and Descartes’ solutions [41].

For some special cases with 1<*p*<2, analytical solutions can be obtained as described in [42]. Finally, for all other *p* values, no analytical solution exists. However, the Newton-Raphson method is more effective in these cases.

For the **f** subproblem, the closed-form solution of Eq (14) can be obtained in the frequency domain:

$$\hat{f}=F^{-1}\left(\frac{F\left(g\right)\circ \bar{F\left(h\right)}+\beta F\left(u_{x}\right)\circ \bar{F\left(\nabla_{x}\right)}+\beta F\left(u_{y}\right)\circ \bar{F\left(\nabla_{y}\right)}}{F\left(h\right)\circ \bar{F\left(h\right)}+\beta F\left(\nabla_{x}\right)\bar{F\left(\nabla_{x}\right)}+\beta F\left(\nabla_{y}\right)\bar{F\left(\nabla_{y}\right)}}\right)$$ (19)

where $\bar{F(\cdot )}$ is the conjugate operation and ∘ is the Hadamard product. Recognizing that the operation in the frequency domain is based on an assumption of a circular shift, the image boundaries should be preprocessed to second-order smoothness, as reported previously [43].

Algorithm 2: Image deconvolution

Input: (1) Observed blurry image, **g**;

        (2) PSF, **h**;

        (3) Regularization coefficient, *α*;

        (4) Convergence criteria, *ε* and *k*;

Output: Latent image **f**;

1: Initialize **f**≔**g**;

2: Initialize *β*≔*α*;

3: for *β<ε* do

4: Solve $\hat{u}$ with **f** from Eq (13), then $u≔\hat{u}$;

**5:** Solve $\hat{f}$ with **h** and **u** from Eq (14), then $f≔\hat{f}$;

**6:** *β*≔*kβ*;

7: end

8: return **f**;

The pseudocode in Algorithm 2 describes the deblurring method. First, the observed image, **g**, is used to initialize **f**; the temporary coefficient, *β*, should be set to a small value. Next, the algorithm updates the intermediate optimal values, $\hat{u}$ and $\hat{f}$, alternately. Meanwhile, *ε* is set to a large value and it is ensured that the condition *k*>1 is met, so that *β*→∞. Finally, the algorithm converges to a stable state, yielding the best approximation of the original image.

### Experimental evaluation

#### Experimental setup

**Fig 8:**
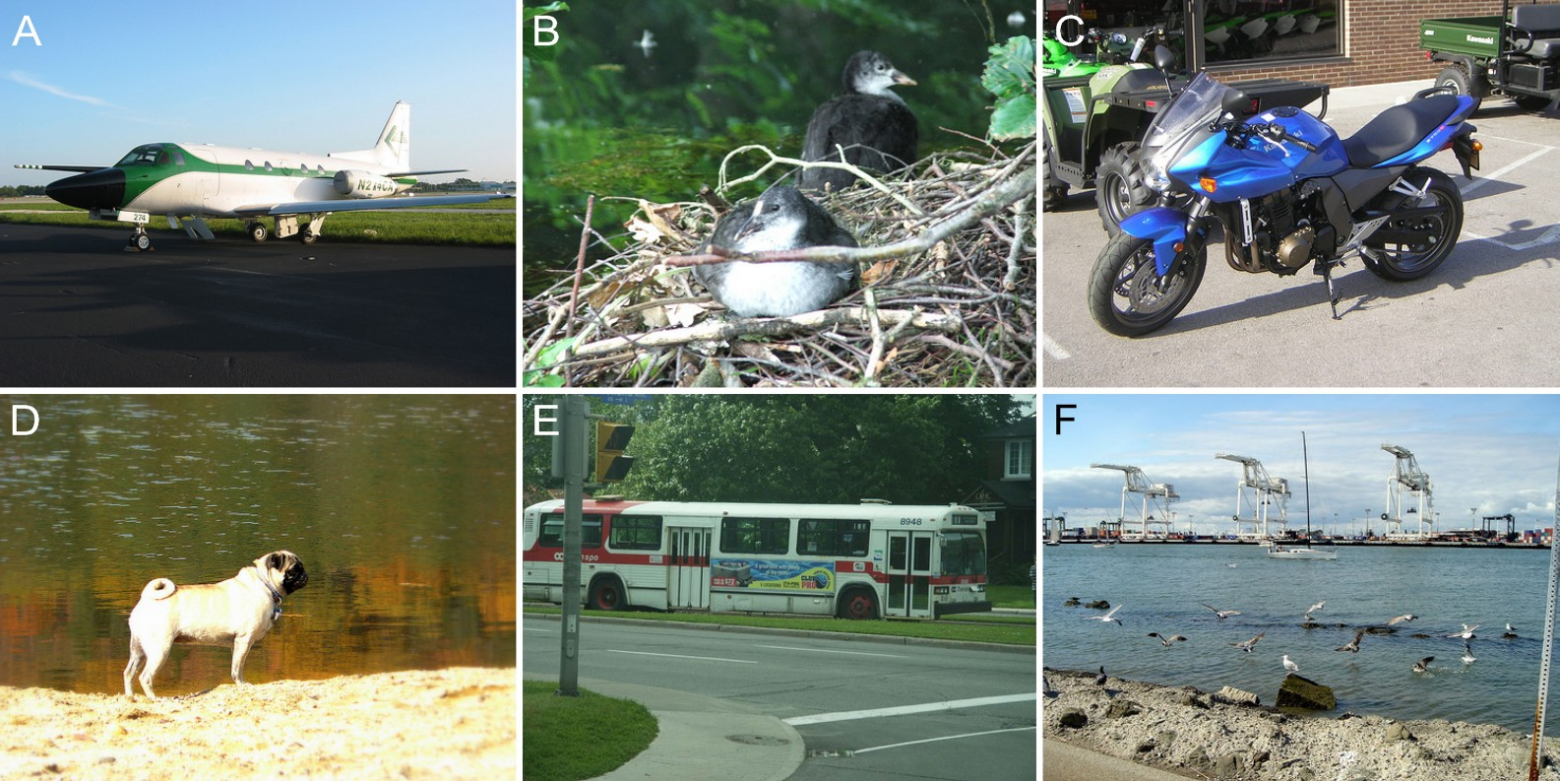
Examples of the pascal VOC dataset.

A training dataset from the Pascal Visual Object Classes (VOC) dataset was used [44]. The dataset consisted of 16,135 natural images of animals, humans, transportation, landscapes, and architectures, among other entities. Fig 8 shows some examples of images from the Pascal VOC dataset. Different artificial blurs were induced in them using increasing parameters so as to build a training set for each blur type, including a series of Gaussian blurs whose *σ* values were increased from 1 to 5 in steps of 0.2 and whose dimensions were of 25×25 pixels. For linear motion blur, estimating the motion length and the orientation simultaneously will lead to overfitting. Thus, in the experiment, we maintained the same orientation and trained a GRNN for each of the five values for the angle *Φ*; i.e., 0°, 30°, 45°, 60°, and 90°, respectively. The motion length, *L*, increased from 2 pixels to 20 pixels.

Next, the blur features, $R_{norm}$, were extracted from each training set. Hypothesis testing [45, 46] was performed to counter the adverse effects of the outliers. Finally, the features and their corresponding parameters were used as the training sets for the GRNNs while setting the spreading rate, *δ*, to 0.6–0.9. The training procedure is similar to that of an RBFN: 1) An unsupervised learning method is needed to determine a set of offsets in the activation function. 2) A least squares method is used to train the weights in the summation before the output. Furthermore, the difference is that each unit of a GRNN is influenced by every sample from the training set. Thus, each training sample *x*_*q*_ acts as the offset of the activation function in each pattern unit of a GRNN. Additionally, in the summation layer, the outputs of the pattern unit *ρ*_*q*_ are weighted with the corresponding values of the training samples, *y*_*q*_, when going to the denominator unit *S*_*D*_, but are weighted with one when going to the numerator unit *S*_*N*_. Because of the use of the one-pass algorithm, the training procedures converged rapidly and stably. The training was implemented in MATLAB using an Intel Xeon E5-2620 v4 CPU (2.1 GHz) and an NVIDIA 1080 GPU. It took approximately 10–15 minutes to generate a network.

As can be seen from the flowchart in Fig 7, for an image blurred by an unknown PSF, the blur feature should be extracted first. Then, using the GRNN as well as the parametric blur models, its PSF can be estimated successfully. In this manner, the latent image can be restored from the blurry image and the PSF estimated by the deconvolution method described in Algorithm 2. The value of coefficient *α* in Eq (12) was determined by trial and error, while the criteria for convergence in the algorithm were set as *ε* = 1*e*4 and *k* = 2, respectively.

**Fig 9:**
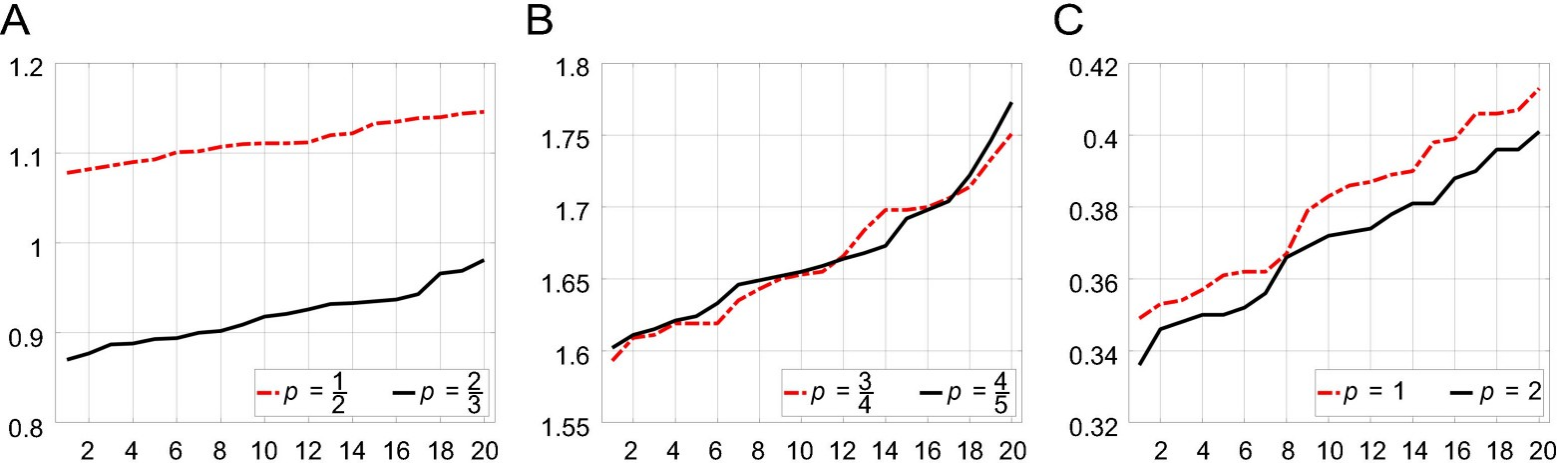
Cost duration for different *p* values. Algorithm 2 was tested for a 768×512×3 image that was blurred by a series of defocus blurs. The *x*-axis represents the radius while the *y* -axis shows the runtime (in seconds).

Next, we evaluated the convergence speed of Algorithm 2 for different *p* values. A series of defocus blurs with increasing radii were simulated in order to test the performance of the solution methods for Eq (14). The simulations were performed on the same platform as that used in the training procedure discussed above. Fig 9 shows the cost times for *p* values of 1/2, 2/3, 3/4, 4/5, 1, and 2. Generally, the Newton-Raphson method is effective for solving Eq (14). However, as discussed in the previous section, for special cases, such as those where *p* = 1/2 and *p* = 2/3, analytical solutions exist, as given in Eq (17) and Eq (18), that converge faster than those for 0<*p*<1 values. This fact is also shown in Fig 9A and 9B. Moreover, when *p* = 1, Eq (14) becomes a one-dimensional shrinkage problem [15] and converges much faster, as shown in Fig 9C. When *p* =2, the image prior reduces to a Gaussian prior, and its deblurred equivalent exhibits a greater number of ringing artifacts and more noise than does the sparse prior [47]. As a result, we used *p* = 1 for the rest of the evaluations and comparisons.

**Fig 10:**
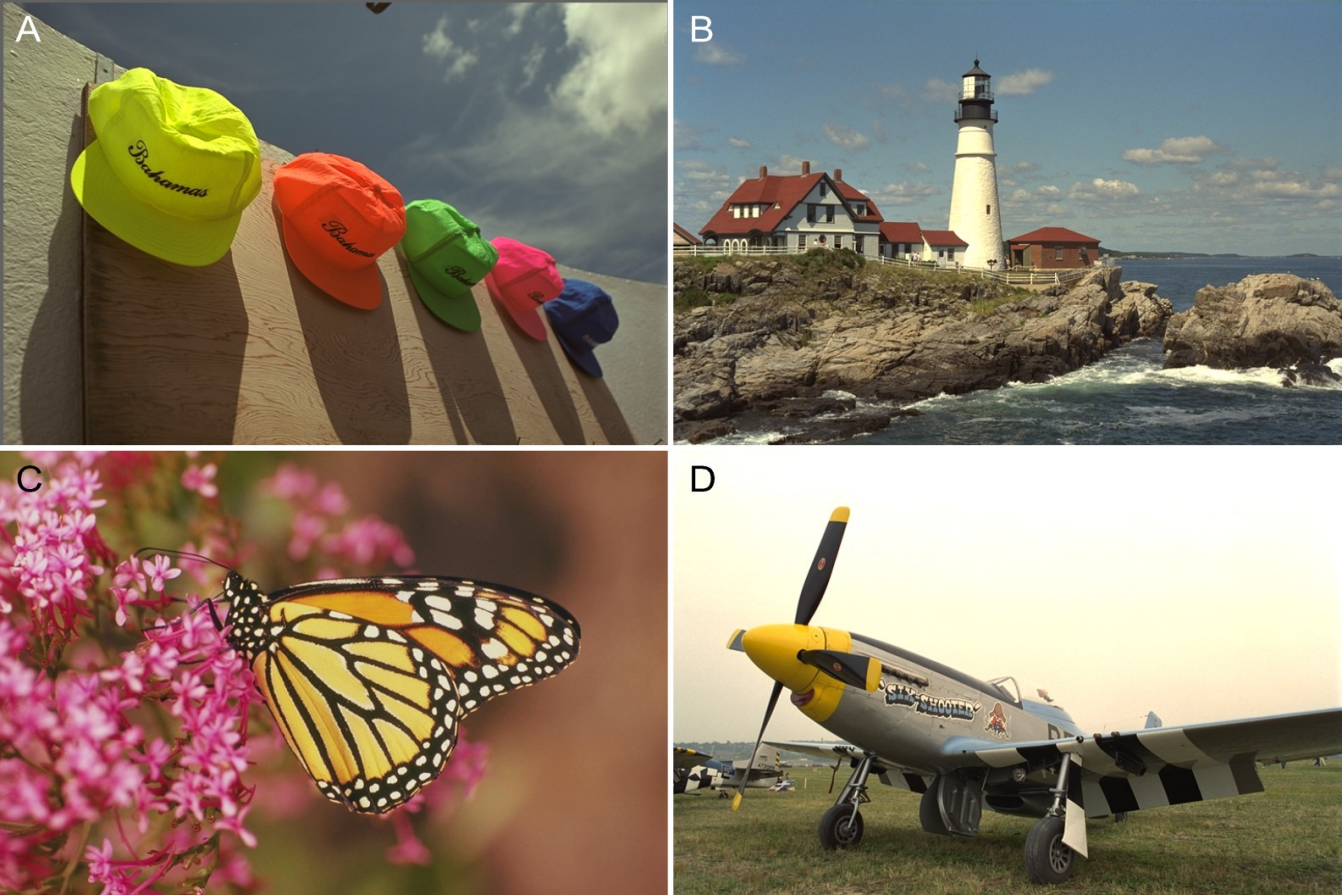
Examples of the ground truth images. (A) “caps.bmp.” (B) “lighthouse2.bmp.” (C) “monarch.bmp.” (D) “plane.bmp.”.

The test images were taken from the LIVE database [48]. In this paper, they are numbered as “Img01,” “Img02,” etc. for ease of comprehension. Table 1 shows their labels and filenames as well as the parameters for the simulated blur models. Fig 10 presents the examples of ground truth images. The testing process is described in the following subsection.

**Table 1:** Test images used and their blur parameters.

No.	Test images	Parameters		
		Motion blur	Defocus blur	Gaussian blur
Img01	bikes.bmp	*L* = 10,*Φ* = 90°	*r* = 4	*σ* = 1.8
Img02	building.bmp	*L* = 8,*Φ* = 0°	*r* = 5	*σ* = 2.2
Img03	buildings.bmp	*L* = 16,*Φ* = 60°	*r* = 6	*σ* = 2.6
Img04	caps.bmp	*L* = 18,*Φ* = 30°	*r* = 7	*σ* = 2.4
Img05	carnivaldolls.bmp	*L* = 18,*Φ* = 45°	*r* = 5	*σ* = 2.0
Img06	cemetry.bmp	*L* = 21,*Φ* = 90°	*r* = 6	*σ* = 2.8
Img07	churchandcapitol.bmp	*L* = 16,*Φ* = 30°	*r* = 4	*σ* = 2.2
Img08	coinsinfountain.bmp	*L* = 15,*Φ* = 45°	*r* = 3	*σ* = 2.6
Img09	dancers.bmp	*L* = 15,*Φ* = 60°	*r* = 5	*σ* = 2.0
Img10	flowersonih.bmp	*L* = 16,*Φ* = 0°	*r* = 3	*σ* = 1.8
Img11	house.bmp	*L* = 17,*Φ* = 90°	*r* = 4	*σ* = 2.4
Img12	lighthouse.bmp	*L* = 19,*Φ* = 30°	*r* = 6	*σ* = 2.8
Img13	lighthouse2.bmp	*L* = 16,*Φ* = 45°	*r* = 8	*σ* = 3.2
Img14	manfishing.bmp	*L* = 25,*Φ* = 60°	*r* = 9	*σ* = 3.4
Img15	monarch.bmp	*L* = 18,*Φ* = 60°	*r* = 4	*σ* = 2.2
Img16	ocean.bmp	*L* = 15,*Φ* = 0°	*r* = 8	*σ* = 3.2
Img17	paintedhouse.bmp	*L* = 8,*Φ* = 90°	*r* = 3	*σ* = 2.4
Img18	parrots.bmp	*L* = 19,*Φ* = 45°	*r* = 9	*σ* = 3.6
Img19	plane.bmp	*L* = 22,*Φ* = 30°	*r* = 8	*σ* = 3.2
Img20	rapids.bmp	*L* = 12,*Φ* = 90°	*r* = 4	*σ* = 2.2
Img21	sailing1.bmp	*L* = 18,*Φ* = 0°	*r* = 9	*σ* = 3.4
Img22	sailing2.bmp	*L* = 17,*Φ* = 30°	*r* = 5	*σ* = 2.2
Img23	sailing3.bmp	*L* = 18,*Φ* = 45°	*r* = 7	*σ* = 2.8
Img24	sailing4.bmp	*L* = 15,*Φ* = 60°	*r* = 6	*σ* = 2.0
Img25	statue.bmp	*L* = 16,*Φ* = 30°	*r* = 3	*σ* = 2.4
Img26	stream.bmp	*L* = 9,*Φ* = 0°	*r* = 7	*σ* = 1.8
Img27	studentsculpture.bmp	*L* = 8,*Φ* = 90°	*r* = 5	*σ* = 2.2
Img28	woman.bmp	*L* = 11,*Φ* = 60°	*r* = 4	*σ* = 2.0
Img29	womanhat.bmp	*L* = 17,*Φ* = 45°	*r* = 8	*σ* = 3.2

We compared the proposed method with several other blind image deblurring methods. Cho and Lee [49] used the shock filter and bilateral filter with multiple orientations of images to enhance the structures, with the aim of ensuring that the histograms of the gradient domain of the reconstructed images were similar to those of the original one. Krishnan et al. [50] proposed an image prior model based on a piecewise function that approximates the statistical characteristics of natural images using a sparsity representation technique. They solved the deblurring problem using an iterative shrinkage-thresholding algorithm. Zhao et al. [51] proposed another PSF-estimating method based on the bilateral filter and shock filter, whereas Wu and Su [52] restored an image by adopting a unified probabilistic model of the image for solving the MAP problem.

To measure the performance of the proposed method and compare it with other similar methods, we used the peak signal-to-noise ratio (PSNR) [53], structural similarity (SSIM) index [53, 54], and feature similarity (FSIM) index [55] as the image quality assessment (IQA) metrics.

## Experimental results

**Fig 11:**
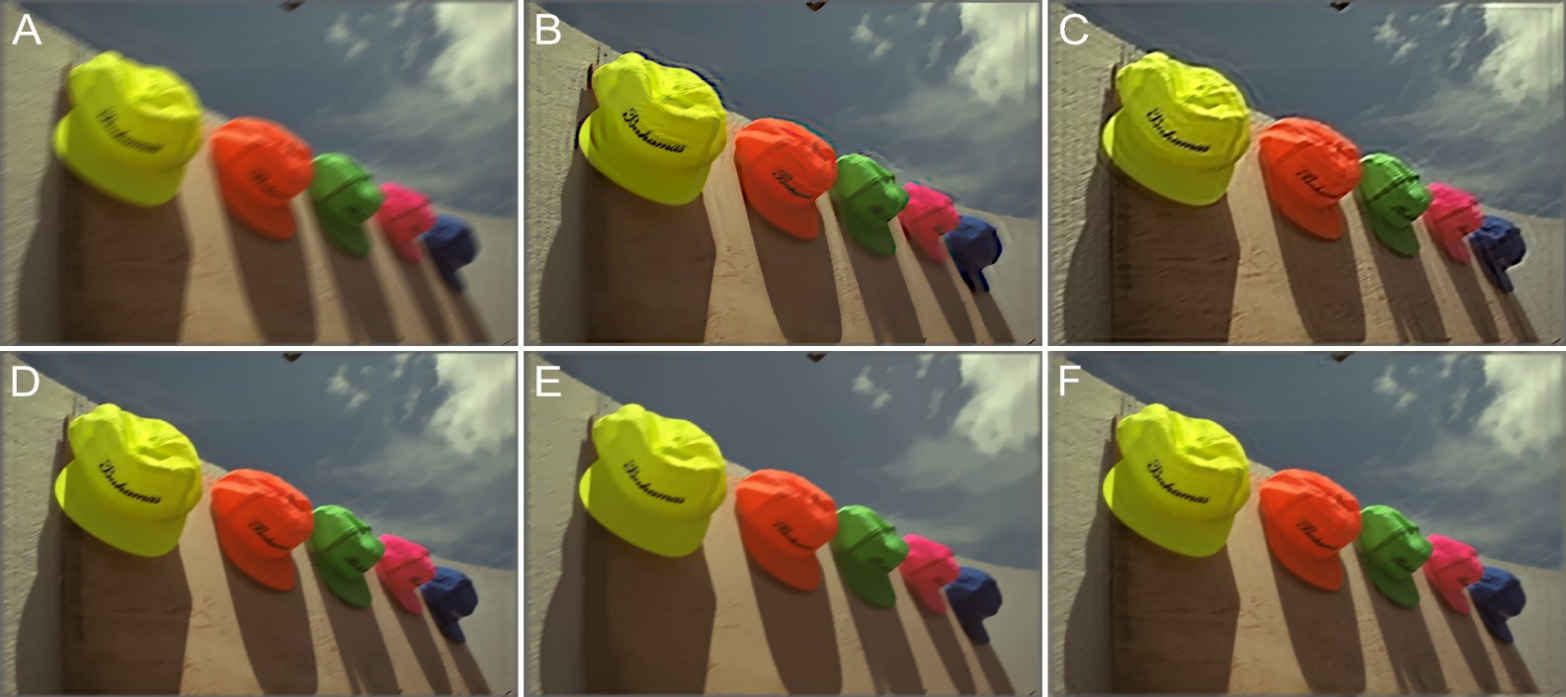
Motion blur with *L* = 18 and *Φ* = 30° for the “caps.bmp” image. (A) Blurred image. (B) Method in [49]. (C) Method in [50]. (D) Method in [51]. (E) Method in [52]. (F) Proposed method.

The test images were blurred by motion blur, defocus blur, and Gaussian blur using the model parameters listed in Table 1. An example of “Img04” blurred by motion blur using (*L*,*Φ*) = (18,30°) and the deblurring results are shown in Fig 11. The brand names written on the caps and the grains in the wood wall behind them are blurred and indistinct in Fig 11A. The deblurring methods described in [49] and [50] introduce ringing effects both around the caps and the image borders, as shown in Fig 11B and 11C. This phenomenon is reduced by the methods described in [51] and [52], as is evident from Fig 11D and 11E. However, the wood grains in the image are not sharp and clear. In contrast, as can be seen from Fig 11F, the proposed method restores both the texture and the background. Table 2 lists the IQA data of a comparative analysis performed on the blurred images.

**Table 2:** IQA data for the comparison results of Figs 11, 12, 14 and 16.

Test images	Parameters	IQA	Method in [49]	Method in [50]	Method in [51]	Method In [52]	Proposed method
caps.bmp	Motion blur *L* = 18 *Φ* = 30°	PSNR	30.964	28.212	28.451	29.768	**31.864**
		SSIM	0.9597	0.9388	0.9413	0.9495	**0.9737**
		FSIM	0.9295	0.9010	0.9091	0.8909	**0.9489**
lighthouse.bmp	Motion blur *L* = 16 *Φ* = 45°	PSNR	23.311	22.610	22.533	23.102	**24.465**
		SSIM	0.8419	0.8347	0.8238	0.8492	**0.8746**
		FSIM	0.8496	0.8518	0.8447	0.8737	**0.8905**
mornach.bmp	Defocus blur *r* = 4	PSNR	25.969	25.123	25.954	25.989	**26.990**
		SSIM	0.9547	0.9484	0.9596	0.9550	**0.9628**
		FSIM	0.9212	0.9166	0.9264	0.9121	**0.9371**
plane.bmp	Atmospheric turbulence blur *σ* = 3.2	PSNR	26.064	25.846	27.189	26.972	**28.030**
		SSIM	0.8865	0.8710	0.9060	0.9020	**0.9343**
		FSIM	0.9039	0.8972	0.9371	0.9244	**0.9508**

**Fig 12:**
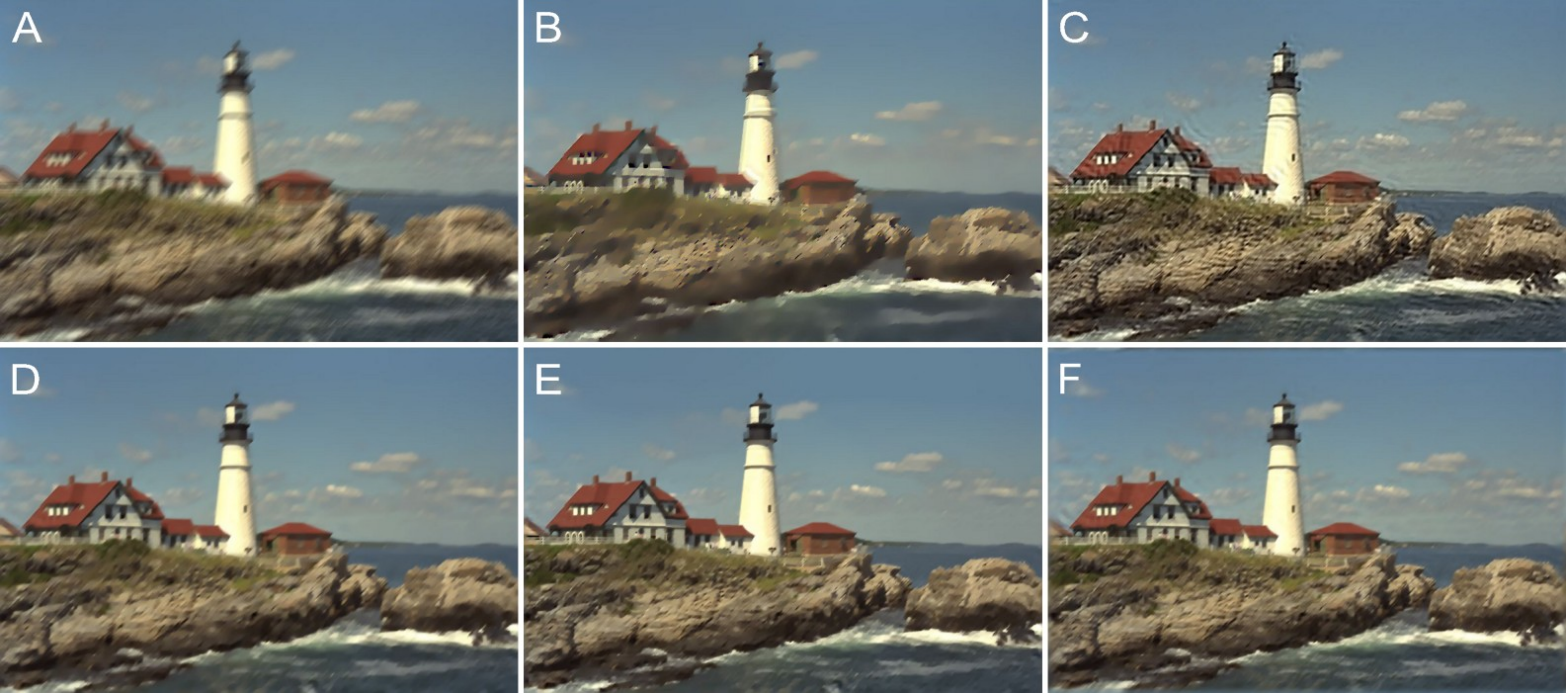
Motion blur with *L* = 16 and *Φ* = 45° for the “lighthouse2.bmp” image. (A) Blurred image. (B) Method in [49]. (C) Method in [50]. (D) Method in [51]. (E) Method in [52]. (F) Proposed method.

**Fig 13:**
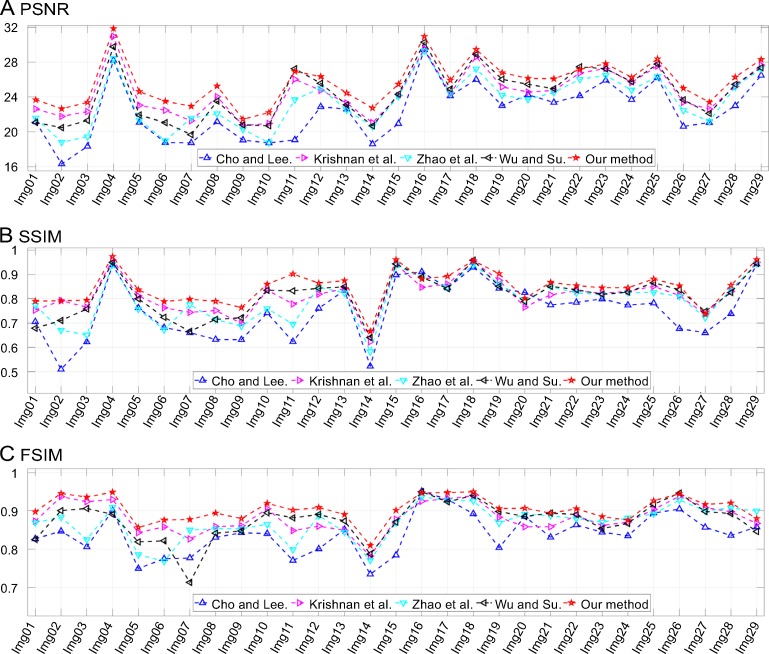
Results of comparisons of test motion-blurred images. The corresponding motion length, *L*, and orientation, *Φ*, values are listed in the third column of Table 1.

Fig 12 shows another example of motion blur with (*L*,*Φ*) = (16,45°); the image here is “Img13.” The lighthouse and cabins are blurred and unclear in Fig 12A. As can be seen from Fig 12B, the deblurring method described in [49] reduced some of the blurriness; however, the visual effect is still lacking. Further, as can be seen from Fig 12C, the method described in [50] restored the sharpness but introduced strong artifacts. These limitations were overcome by the methods described in [51] and [52] (see Fig 12D and 12E, respectively) as well as by the proposed method (see Fig 12F), which restored most of the details. The statistics related to the IQAs as determined from a comparative analysis of the motion-blurred images are shown in Fig 13. For both examples, namely, “Img04” and “Img13,” the PSNR, SSIM, and FSIM were the highest for the proposed method. In addition, Table 2 lists the original IQA data.

**Fig 14:**
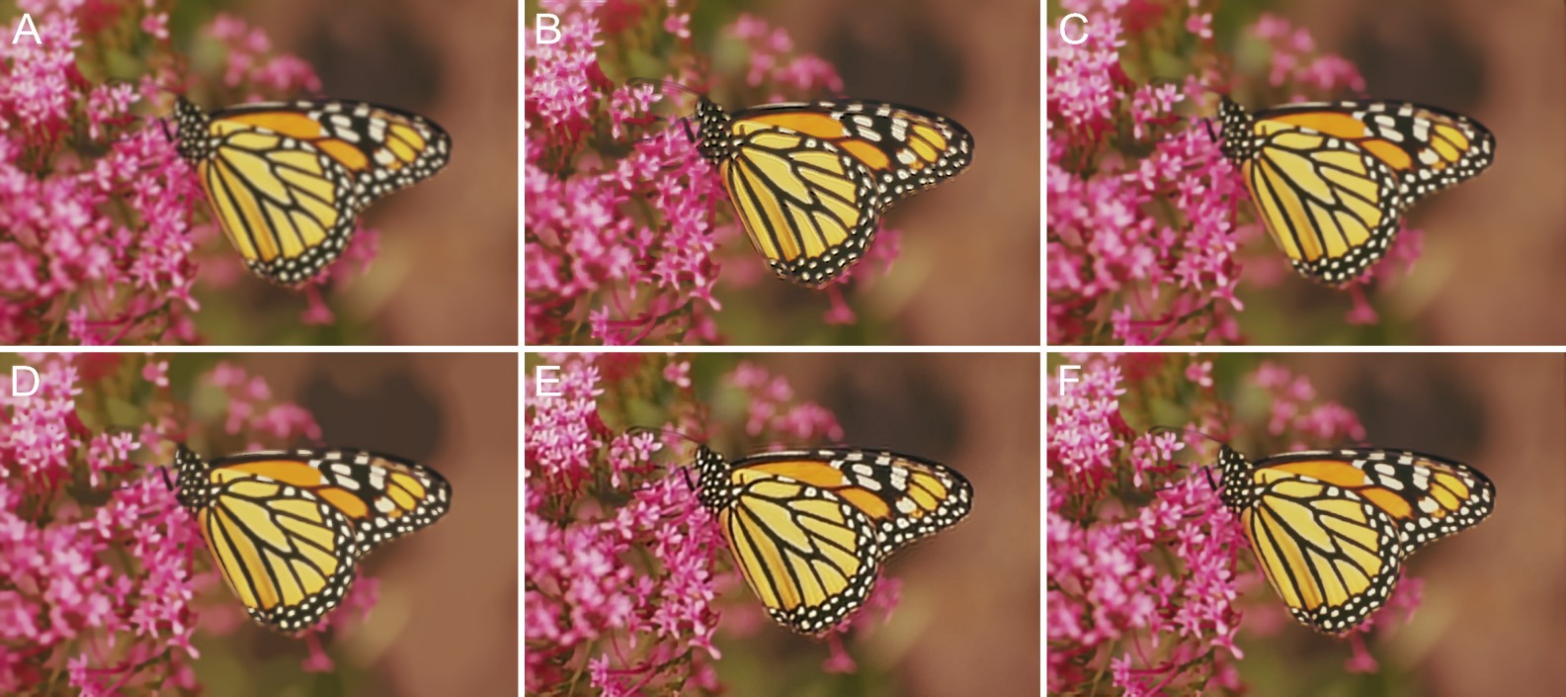
Defocus blur with *r* = 4 for the “monarch.bmp” image. (A) Blurred image. (B) Method in [49]. (C) Method in [50]. (D) Method in [51]. (E) Method in [52]. (F) Proposed method.

**Fig 15:**
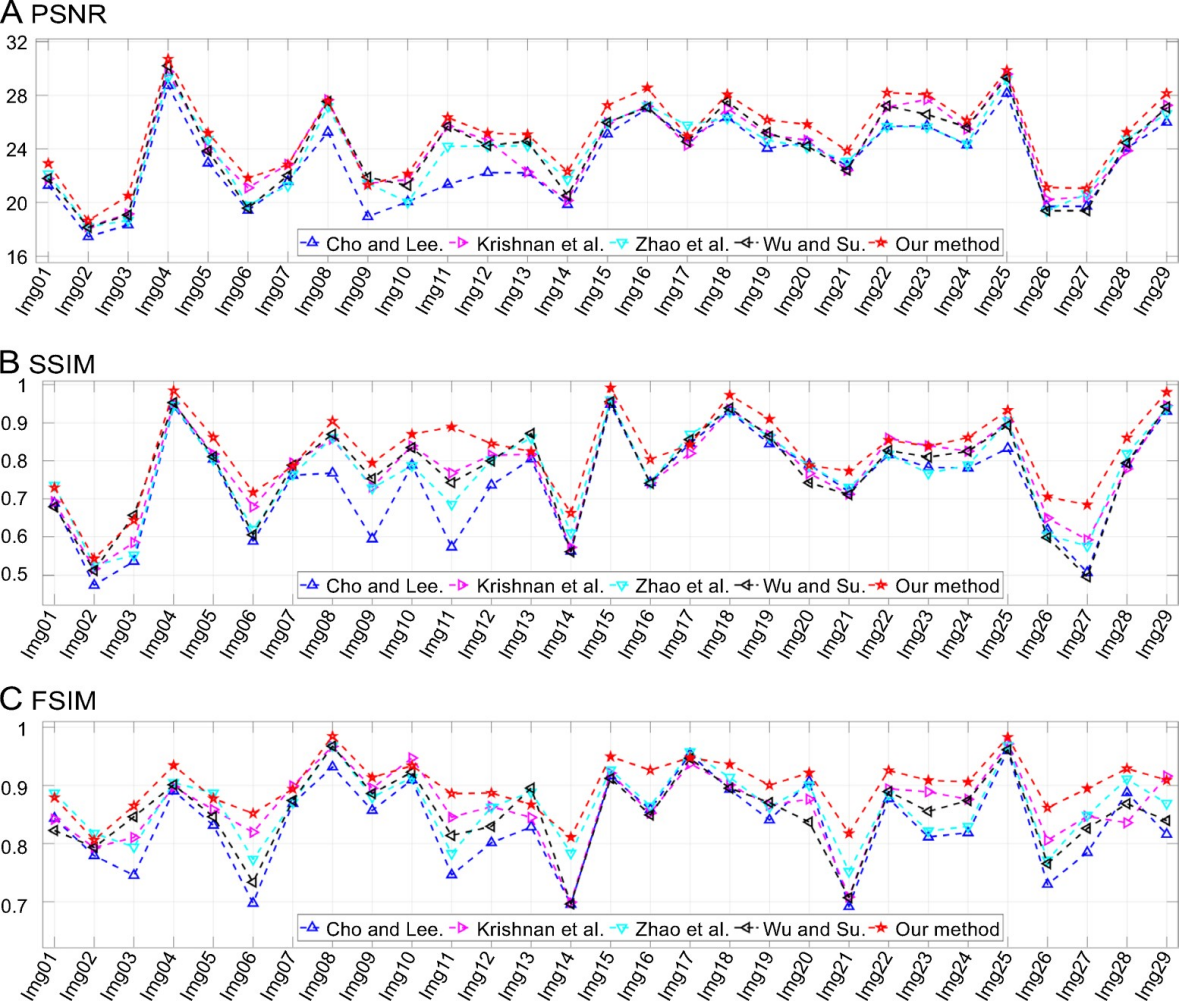
Results of comparisons of test defocus-blurred images. The corresponding radius, *r*, values are listed in the fourth column of Table 1.

Similarly, Fig 14 shows another example, namely, “Img15,” which was blurred by defocus blur with *r* = 4. In daily life, photograph degradation resulting from the camera being out of focus is a common phenomenon. As can be seen from the figure, the texture on the wings are blurred and fuzzy, although the extent of the blurriness is low. The compared methods are all able to restore the details effectively, but our method has the least ringing effect. It should be noted that there are spatially varying blurs in part of the ground truth image, such as the background and some of the flowers; consequently, these details cannot be restored. Nonetheless, in the case of the “mornach.bmp” image, the proposed method outperformed the others, as is evident from the IQA data obtained from a comparison of the defocus-blurred images (see Table 2 and Fig 15).

**Fig 16:**
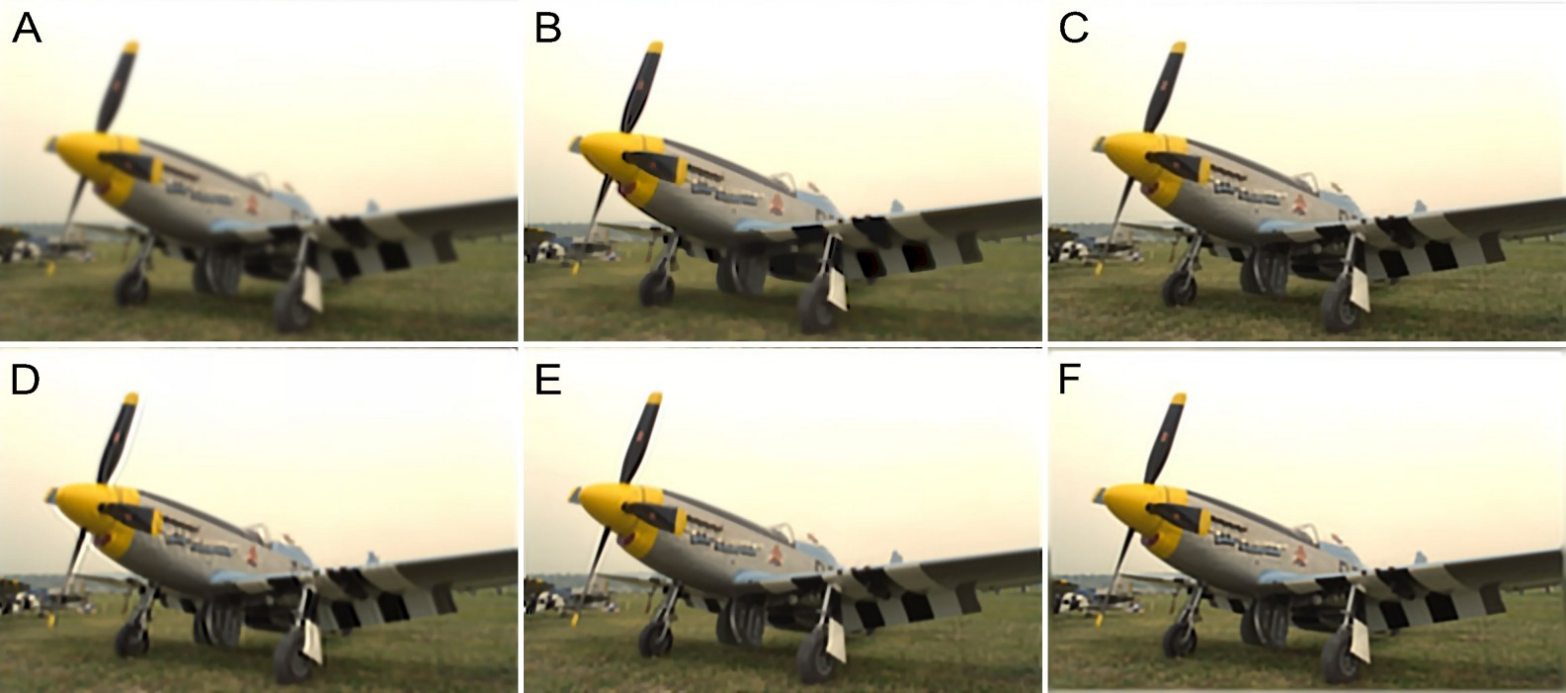
Atmospheric turbulence blur with *σ* = 3.2 for the “plane.bmp” image. (A) Blurred image. (B) Method in [49]. (C) Method in [50]. (D) Method in [51]. (E) Method in [52]. (F) Proposed method.

**Fig 17:**
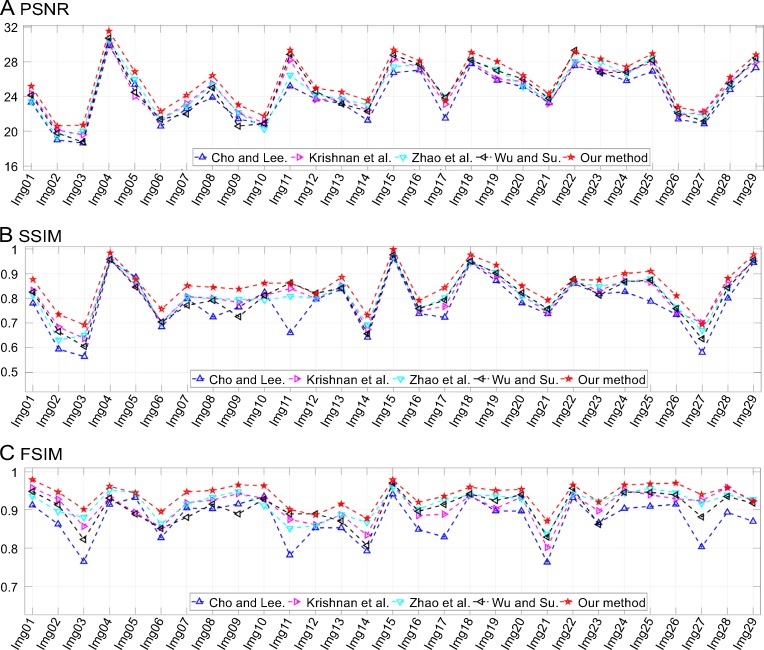
Results of comparisons of test atmospheric turbulence-blurred images. The corresponding parameter, *σ*, values are listed in the fifth column of Table 1.

In Fig 16, an image, namely, “Img19,” blurred by Gaussian blur with *σ* = 3.2 is shown. The outline of the plane is unclear and so are the words on the fuselage (see Fig 16A). Atmospheric turbulence is a complex phenomenon that has troubled scholars for decades. Its PSF is symmetrical and its support domain is infinite both in the spatial and frequency domains. In practice, truncation in the spatial domain makes the deblurring procedure feasible. However, it leads to unavoidable errors that reduce the accuracy of the restoration results. As shown in Fig 16B–16F, all the methods could recover the outline of the plane successfully but were incapable of restoring the words on the fuselage. Their quality assessments are listed in Table 2. It can be seen from the results that the proposed method outperformed the others. In addition, Fig 17 shows the IQA parameters for all the tested images. It can again be seen that the proposed method was superior to the other methods in most cases.

In addition, the comparative analysis showed that ringing effects are inevitable in the case of the conventional blind blur removal method [49, 50]. Further, while the ringing effects were reduced to a certain extent in the cases of the methods given in [51] and [52], they did exist. On the other hand, the proposed deblurring method exhibited promising results in terms of both the visual quality and objective assessment parameters.

**Fig 18:**
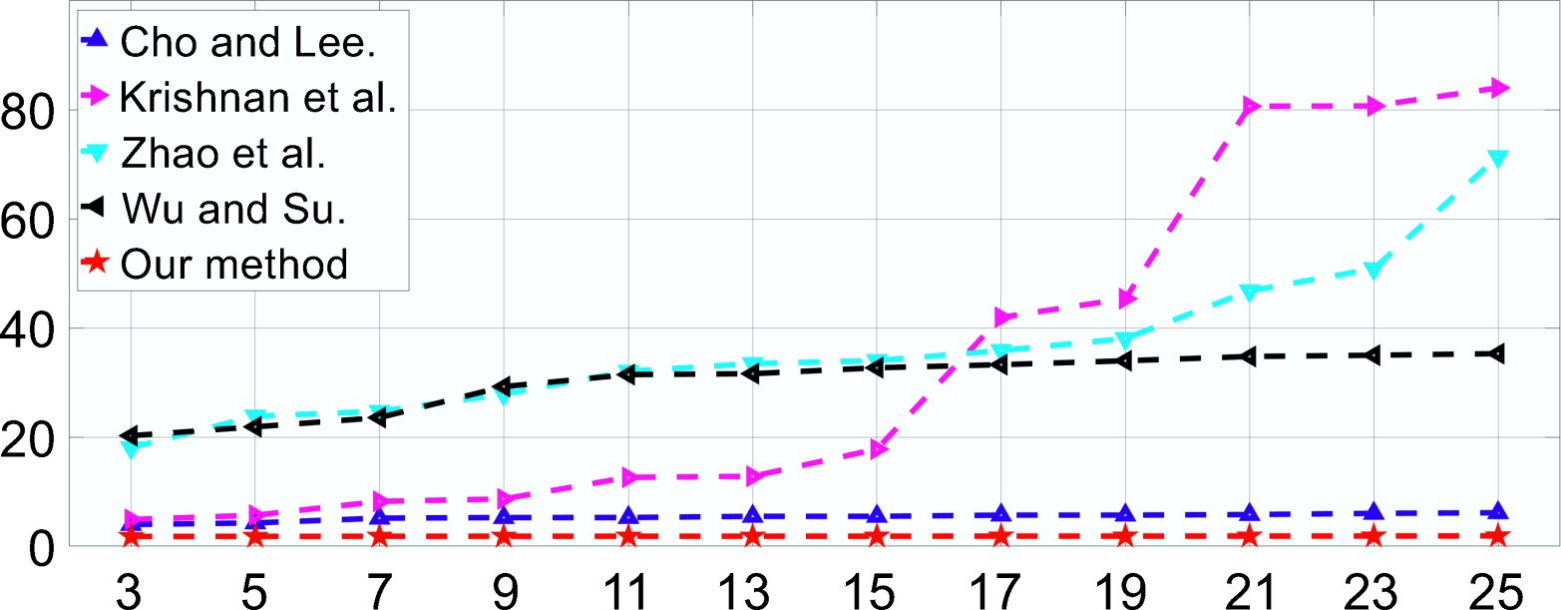
Results of average processing speed for the compared methods. The *x*-axis lists the PSF sizes, which range from 3×3 to 25×25, while the *y*-axis shows the total runtime (in seconds).

Fig 18 compares the proposed method with the other methods in terms of speed. The size of the testing images was 768×512×3, and the sizes of the testing PSFs ranged from 3×3 to 25×25. As can be seen, the processing time of the methods in [50–52] increases rapidly with the PSF size. The method in [49] converges rapidly, but the IQAs of its results are unsatisfactory. Our method requires the lowest runtime and produces a deblurring result within five seconds, whereas tens of seconds or even more than a minute are required by the other methods.

### Real-life applications

**Fig 19:**
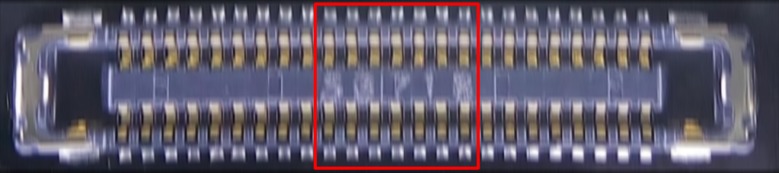
Motion-blurred photograph in real-life application.

**Fig 20:**
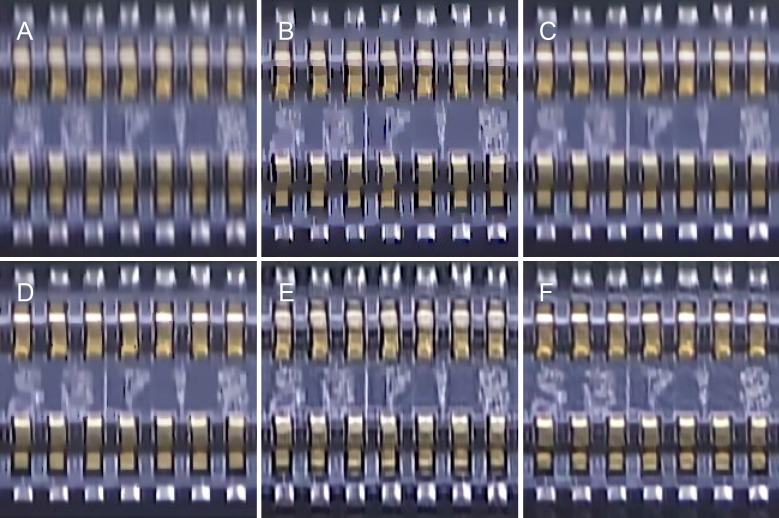
Results of comparisons with Fig 19. (A) Blurred image. (B) Method in [49]. (C) Method in [50]. (D) Method in [51]. (E) Method in [52]. (F) Proposed method.

In the electronics industry, integrated circuit boards moving on a conveyor line are examined by a fixed-position camera hanging over them. As a result, these boards suffer from a significant degree of motion blur and their photographs are of extremely poor quality (see Fig 19 and Fig 20A). The deblurring results from the methods in [49–52] are shown in Fig 20B–20E. As the movement of the conveyor can be regarded as linear motion with a constant orientation, its PSF can be estimated using the proposed method. The thus-obtained deblurring results are shown in Fig 20F. It can be seen clearly that the text is clearer and the area around the pins of the chip shows more details.

## Conclusions

In this study, a blur parameter estimation technique based on deep learning and a nonlinear regularized deblurring method based on sparse representation for image blur removal were proposed and evaluated. The blur features were determined based on the autocorrelation of the gradient domain of the image, which is strongly related to the blur degree. Further, a GRNN was adopted for learning-based parameter estimation. More than ten thousand natural images were used to generate training samples of motion blur, defocus blur, and atmospheric turbulence blur. Using the GRNN, the PSF of the test blurry images could be estimated correctly. Moreover, the deblurring algorithm converged quickly owing to the use of a half-quadratic method. The results of simulations and a comparative analysis confirmed that the proposed method is superior to other deblurring methods. The main disadvantage of GRNN is that its size can be huge, which results in expensive computation. Another limitation of the proposed method is that it can only deal with spatially invariant blurs in specific scenarios. Although the training process is time-consuming, it only needs to be carried out once. In future work, a better training dataset and blur features will be considered to improve the performance of the neural network, and the deblurring model can be extended into nonlinear blurs for wider application.
